# Three Surgical Cases

**Published:** 1892-12

**Authors:** Erastus E. Case

**Affiliations:** Hartford, Conn.


*Second Day—Evening Session.*

Wednesday, June 22d, 8 P. M.

### BUREAU OF SURGERY.

T. M. Dillingham, M. D., *Chairman.*

---

### THREE SURGICAL CASES.

Erastus E. Case, M. D., Hartford, Conn.

---

### Ingrowing Toe-Nails.

I. October 10th, 1889.—Mr. L., a printer, aged twenty years. Ingrowing nail on the right large toe.

The toe is swollen and ulcerating around the margin of the nail.

All the nails of hands and feet are thick and brittle.

The skin on the hands is very dry. In cold weather the skin of the hands cracks so deeply as to bleed.

Slight injuries of the skin ulcerate a long time.

The sweat of the feet is profuse and offensive. ℞ *One dose Graph.*$^{200}$.

October 24th.—The toe is healed. It has given him no trouble since, although it had been an annoyance for several years previous to the prescription. It is useless to state that his general condition was also improved.

II. November 12th, 1890.—Miss N., a superannuated school teacher, aged seventy-seven years, tall and slender, applied for surgical relief from ingrowing nails on both feet. She has been unable to wear shoes for several months, and in consequence has not ventured upon the street. The nails of the toes are thick and look like horns. There is redness and inflammation about the margins of even those nails which are not ingrowing. ℞. *One dose Magnes-p. Aus.*$^{20m}$ (*F.*).

November 19th.—The redness about the margin of the nails is gone, some soreness, however, still remains. *No medicine.*

December 11th.—The soreness is entirely gone. She has had no trouble from the nails since that time.

### Fistula Ani.

III. September 22d, 1890, Mr. S., a lawyer, unmarried, aged forty-seven years, of a studious, sedentary habit, with black eyes, black curly hair and clear white skin, consulted me regarding an anal fistula. He would not permit the doctors to cut it open, as they wished to do. He had therefore suffered on without treatment, steadily growing worse for several years. He came to me "because he thought I was less fond of using the knife than were the allopathic doctors." And as he said, "Something must be done or he would soon be unable to carry on his professional work."

The condition is thus described:

At the right of the anus is a swelling in the form of an ellipse, with diameters of one and two inches, containing fluid. This is quite sore and becomes inflamed if he sits long continuously. At its posterior margin is a small opening from which an offensive pus escapes. From this swelling, or cesspool as I will name it, there are three burrowing sinuses. The first formed and longest extends forward across the perineum and ends upon the posterior surface of the scrotum, a distance of five inches. The second is four inches long and extends outwardly upon the buttock at right angles to the perineal raphé. The third and last formed sinus, which began two months ago, is two and a half inches in length, situated between the other two, and forms a curve tending toward the perineum. These sinuses feel like whip-cords beneath the skin. They have very little soreness and no external openings. Aside from lack of exercise the habits of the patient are beyond criticism. All the bodily functions are carried on in a normal manner. ℞ *Twelve powders Sil.*$^{200}$*, four doses per day.*

October 2d.—The swelling has less soreness and prominence. The discharge has already become less offensive. *No medicine.*

October 16th.—The original cesspool is smaller and now discharges an inoffensive, watery fluid. The longest sinus is discharging at its anterior extremity on the back of the scrotum. *No medicine.*

October 31st.—A watery discharge oozes from the end of the sinus as well as from the cesspool, which is not more than half the size which it had originally. *One dose Sil.*$^{40m}$ *(F.).*

November 24th.—Less discharge now from the sinus. The cesspool fills and discharges about once a week. *No medicine.*

December 15th.—The discharge from the sinus is now very slight. The original opening discharges frequently and a lessening quantity at each time. *One dose Sil.*$^{72m}$ *(F.).*

January 15th, 1891.—There have been only two discharges from the cesspool in a month. *No medicine.*

February 10th.—The longest sinus has stopped discharging, and the other two have opened at their extremities. *One dose Sil.*$^{cm}$ *(F.).*

March 13th.—The discharge is lessening at all points. The sinuses are losing their hardness. *No medicine.*

April 14th.—Improvement is going on steadily. *No medicine.*

May 11th.—An increase in the amount of the discharge has been noticed for a few days. *One dose Sil.*$^{cm}$ (*Skinner*).

June 6th.—The cesspool has entirely disappeared.

Only a few drops of watery fluid escape from the original opening on any day. On some days none at all.

A slight discharge from the last two sinuses. *No medicine.*

November 2d.—Occasionally some discharge from the original opening, but none from the sinuses. No trace remains of the longest sinus. *One dose Sil.*$^{M}$ (*F.*).

March 26th, 1892.—No traces of the sinuses or cesspool can be found. Occasionally a little moisture oozes from the original opening. *One dose Sil.*$^{5\,cm}$ (*F.*).

May 12th.—There has been no discharge from the fistula for several weeks.

### Discussion.

Dr. Kent—The application of a remedy for ingrowing toe-nails is an inference from the proving. The remedy probably did not produce ingrown toe-nails, but rather a sensation as if the nail were growing in, and this paper is a verification of the truth and genuineness of the proving. The remedy has cured it in so many instances that there can be no doubt about its effect in that trouble. I remember the first time I used it was in a patient who had no other symptoms. He had the shoe cut over the toe to prevent the pressure of the upper upon it. On examination I found an ingrown toe-nail that had been very much treated. He had scraped it and stuffed cotton under it, and so on, without a cure resulting. I gave him a single dose of the Polus Australis, and to my astonishment in two or three days he told me that it had taken away the pain, and it finally cured him.

Dr. F. Powel—I should like to add my testimony to the efficacy of this remedy on ingrowing toe-nails. I have had a number of cases, probably as many as twenty, cured by it. Only two weeks ago the son of one of my colleagues in Chester had a trouble of this kind with his toe of a very severe nature. It gave immediate relief. My success with it has been almost unvarying. It acts very promptly.

Dr. M. Powel—A friend of mine showed me a thumb the nail of which was ingrown and very painful. I gave him one dose on the tongue of Magnes-aust.$^{10M}$, and when I met him again afterward he said it was well.

Dr. M. F. Taft—I was called to see one of the Sisters in a Catholic convent on account of badly ingrown toe-nails. She was very far gone in consumption. I did not take the totality of the symptoms. Knowing the reputation of the remedy in that trouble, I gave it as an experiment. In one week the toes were greatly improved, and in two weeks were well, but I have always wondered whether the patient was injured by it, although I did not notice any aggravation. This idea was raised by the rapid healing of the toes without other improvement.

Dr. Reed—Some seven or eight months ago I read an article in the *Medical Advance* by Dr. Powel on this remedy for ingrowing toe-nails, and I sent to him for some of it, because I was suffering myself from such a complaint. I took his medicine and my toe-nails are just as bad as ever.

Dr. Kent—Dr. Reed has a lot of other symptoms that should be prescribed for instead of the toes. He knows that as well as anybody here.

Dr. S. Long—I have had in the last two or three months two cases of ingrowing toe-nails. They were cured, but not with one remedy. I cured the patient with the toe-nails.

Dr. E. E. Case—There is no specific for ingrowing toe-nails. I have cured them with Graphites and with Silica.

Dr. A. Campbell—Is there any peculiar appearance of the nail when the Polus Australis is indicated?

Dr. Case—None that I know of.

